# Nutrition in Liver Cirrhosis and Transplantation—Current State and Knowledge Gaps

**DOI:** 10.3390/nu12030680

**Published:** 2020-03-03

**Authors:** Maitreyi Raman, Puneeta Tandon, Manuela Merli

**Affiliations:** 1 Division of Gastroenterology, Department of Medicine, University of Calgary, 6D33 TRW Building, Calgary, AB T2N 4N1, Canada; 2 Department of Medicine, University of Alberta, Edmonton, AB T6G 2R3, Canada; ptandon@ualberta.ca; 3 Gastroenterology, Department of Clinical Medicine, Sapienza University of Rome, 00185 Rome, Italy; manuela.merli@uniroma1.it

Cirrhosis of the liver is a leading cause of morbidity and mortality [1]. Awareness of the importance of nutrition in the management of cirrhosis is critical to help improve clinical outcomes in this frequently frail patient population. There are several established roles for integrating nutrition into clinical care pathways in cirrhosis care, such as routine nutritional screening and assessment, and providing optimized energy and protein dosing [2]. However, several gaps in meeting guideline-based recommendations remain. For example, the ideal tools for calculating resting energy expenditure are unknown, as predictive equations to calculate energy requirements are inaccurate compared to gold-standard indirect calorimetry, which is not practical in the clinic-based setting. Several nutrition screening tools are available; however, very few of these have been validated against nutrition assessment tools, or relevant clinical outcomes [3]. A busy clinical setting may limit clinicians from engaging in nutrition screening or assessment due to a lack of knowledge about optimal screening tool selection. Sarcopenia is a well-established risk factor for adverse outcomes in cirrhosis; however, identifying this in clinical practice remains challenging.

This Special Issue of *Nutrients*, “Nutrition in Liver Cirrhosis and Liver Transplantation”, includes 14 manuscripts dedicated to exploring the spectrum of nutrition relevant to these topics, both adding to the existing literature in established areas and including novel pilot discoveries for further investigation. Two original manuscripts are specifically focused in weight gain and obesity risks in the post-liver transplantation setting [4,5]. Metabolic disorders were observed as a frequent complication after liver transplantation, present in up to one third of patients. Sarcopenia was found to be worse in obese individuals and found to be associated with hepatic encephalopathy. Four studies investigated the effects of nutrient supplements (docosahexaenoic acid, B-Hydroxy-B-Methyl Butyrate, and branched-chain amino acid alone, or synbiotics delivered together with branched chain amino acids) on various outcomes (portal hypertension, muscle mass and functioning, glucose metabolism, and hepatic encephalopathy, respectively) in both cirrhosis and post-liver transplantation [6,7,8,9]. While sarcopenia was not linked to hospital admissions or death in patients with medical refractory ascites, it was strongly associated with increased episodes of acute kidney injury [10]. The prognostic ability of nutrition risk assessment tools was further investigated. The modified NUTRIC (Nutrition Risk in Critically ill) score in cirrhosis patients with acute variceal bleeding demonstrated good discriminative power to predict 6-week mortality [11]. The final original manuscript investigated the accuracy of handheld indirect calorimetry compared to the gold standard Vmax Encore metabolic cart to predict resting energy requirements [12]. Study results identified a wide variability of resting energy expenditure between the handheld device and the metabolic cart at an individual level, suggesting the need for more accurate and feasible methods of determining resting energy expenditure in cirrhosis patients to guide personalized energy dosing. 

Five review manuscripts were included in this Special Issue. First, a systematic review explored the accuracy of predictive energy expenditure equations in cirrhosis, identifying their limited accuracy by frequently underestimating requirements [13]. The controversies in diagnosing sarcopenia in cirrhosis were subsequently discussed, creating an action call for standardized sarcopenia definitions to support clinical implementation [14]. The concept of frailty, and its links with sarcopenia and malnutrition, as a relevant factor in cirrhosis outcomes is discussed [15]. Although a growing body of literature has identified the need for nutritional assessment in cirrhosis, food intake analysis still remains a neglected step in nutritional assessment, leading to recommendations for effective individualized educational programs for patients [16]. Finally, the challenges of obesity in the liver transplant setting, including cardiovascular risk assessment and perioperative respiratory and infectious complications, are discussed, with treatment approaches designed to spare further muscle loss [17]. 

These important contributions will provide a platform for future discovery, and will advance the thoughtful discussion of nutrition priorities and clinical integration to optimize patient care and clinical outcomes. 
